# Correction for Estaki et al., “Physical Activity Shapes the Intestinal Microbiome and Immunity of Healthy Mice but Has No Protective Effects against Colitis in MUC2^−/−^ Mice”

**DOI:** 10.1128/mSystems.01096-20

**Published:** 2020-11-03

**Authors:** Mehrbod Estaki, Douglas W. Morck, Sanjoy Ghosh, Candice Quin, Jason Pither, Jacqueline A. Barnett, Sandeep K. Gill, Deanna L. Gibson

**Affiliations:** a Department of Biology, Faculty of Science, University of British Columbia, Kelowna, British Columbia, Canada; b Department of Pediatrics, University of California San Diego, La Jolla, California, USA; c Department of Biological Sciences, University of Calgary, Calgary, Alberta, Canada; d School of Population and Public Health, University of British Columbia, Vancouver, British Columbia, Canada

## AUTHOR CORRECTION

Volume 5, no. 5, e00515-20, 2020, https://doi.org/10.1128/mSystems.00515-20. Page 1: The author byline should appear as given in this correction.

Page 17: The following should be added to the end of the first paragraph of Acknowledgments: “…design, S.G.; analysis, S.G.; expertise, S.G.; approval of the final manuscript, S.G.”

Page 17: The following should be added to the end of the second paragraph of Acknowledgments: “S.G. was a Michael Smith Foundation for Health Research Scholar funded by a Canadian Association of Diabetes Grant in Aid and an NSERC Discovery Grant.”

